# Strengthening Medical Genetics and Genomic Medicine in Colombia: Progress, Challenges, and Strategic Opportunities

**DOI:** 10.1007/s12687-025-00826-y

**Published:** 2025-08-15

**Authors:** Paola Liliana Páez Rojas, Lina María Mora, Juan Sebastián Rincón, Ignacio Zarante Montoya, María Camila León-Sanabria, Ana María Urueña-Serrano

**Affiliations:** 1 Faculty of Medicine, Human Genetics Institute, Pontificia Universidad Javeriana, Bogotá, D.C Colombia; 2 Colombian Association of Medical Geneticists and Genomic Medicina (ACMGen), Bogotá, D.C Colombia

**Keywords:** Genetic services, Medical genetics, Health care facilities workforce and services, Genomics, Education, Patient advocacy

## Abstract

Colombia, an upper-middle-income country with over 52 million inhabitants, has made significant progress in consolidating medical genetics as a clinical specialty, with a growing presence in healthcare system, public health and academia. The development of specialized training programs, the establishment of a professional association, and the inclusion of genetic tests and treatments for rare diseases (RDs) within the health system have been key achievements. Birth defects (BD) remain one of the leading causes of infant morbidity and mortality, and alongside RDs, are recognized as public health priorities. Among regulatory milestones, the Newborn Screening (NBS) Law has strengthened early diagnosis efforts. Nevertheless, operational challenges persist, particularly in the nationwide implementation of genetic services, which remain concentrated in urban centers, creating significant gaps in rural areas. While technologies such as next-generation sequencing (NGS) are increasingly available in the private sector, a persistent fragmentation between molecular diagnosis and clinical care limits their impact. Furthermore, interoperability with health information systems is limited, and the country’s low density of medical geneticists restricts service availability. The Colombian experience underscores the value of institutional coordination, investment in diagnostic infrastructure, and the active role of patient organizations. Despite existing challenges, regional cooperation within Latin America emerges as a strategic opportunity to strengthen medical genetics and expand access for populations affected by RDs and complex genetic conditions.

## Introduction

Colombia, situated in the northwest of South America, Colombia is home to over 52 million people and operates a mixed healthcare system founded on universal coverage through the General System of Social Security in Health (SGSS) (DANE 2024). This system functions via solidarity-based financing, channeling resources from contributors to subsidized individuals within a network of both public and private services providers. While achieving coverage exceeding 95% of the population, challenges persist in ensuring equity, quality, and access, particularly in rural regions (Fitzgerald 2023).

Building upon the 2003 World Health Organization (WHO) regional consultation advocating for state investment, research, education, professional training, and intersectoral coordination in medical genetics (MG) across Latin America (Kofman-Alfaro & Penchaszadeh 2003), this paper examines the progress and ongoing challenges of MG in Colombia. Our focus encompasses regulatory developments concerning rare diseases (RDs), the strengthening of genetic services, the availability of therapies, human resource training, genetic epidemiology (including birth defects, newborn screening, and the registration and follow-up of rare diseases), the integration of genomics into the healthcare system, and the crucial role of patient advocacy.

## Legislative milestones in rare disease care in Colombia

Over the past two decades, the care of patients with rare diseases (RDs) has advanced significantly, with the European Union (EU) at the forefront of these improvements. The EU has sought to integrate the efforts of its member countries on multiple fronts, including promoting the visibility of these diseases and highlighting their impact on public health. An analysis of academic publications over the past 18 years revealed that 23 countries worldwide, including Colombia (Khosla & Valdez 2018) have addressed the issue of RDs in their national plans, policies, or legislation.

**Fig. 1:**
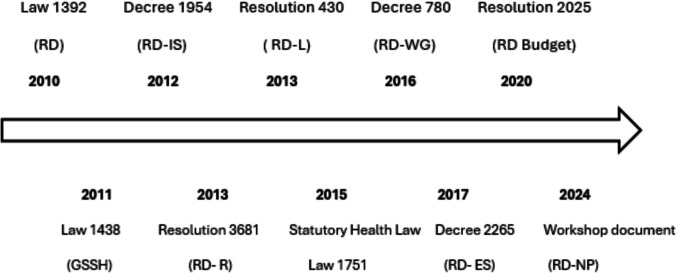
Evolution of health legislation and policies in medical genetics. Source: Author's elaboration. RD: Rare diseases; IS: information system; L: list; WG: working group; R: registry; ES: epidemiological surveillance.; NP: National Plan; GSSH: Law for the Reform of the General System of Social Security in Health

The following section outlines the main legislative developments in the construction of a care framework for individuals undergoing the diagnostic or therapeutic journey for RDs in Colombia. See Fig. 1.

*Law 1392 of 2010* marked a pivotal initial step in establishing a healthcare framework dedicated to the identification, recognition, diagnosis, and treatment of diseases deemed to be of public health interest due to their low prevalence coupled with high diagnostic and therapeutic costs. This legislation defined RDs within the Colombian context as chronic, debilitating conditions posing a significant risk to patients and affecting 1 in 2,000 individuals or fewer. This prevalence threshold was subsequently revised by *Law 1438 of 2011,* which adjusted the cutoff to *1 in 5,000 individuals*. The law further categorized these conditions into rare, ultra-rare, and neglected diseases—the latter understood as prevalent in developing countries, disproportionately affecting the poorest populations, and lacking effective, appropriate, and accessible treatments.

Building upon this legislative foundation, the groundwork was laid for the development of clinical care guidelines, the creation of a list of diseases eligible for benefits and monitoring, the definition of diagnostic criteria and tests, the promotion and implementation of research initiatives, and the establishment of awareness and dissemination strategies concerning the impact of timely diagnosis and intervention. The regulation also proposed the creation of a national unified patient registry *(National Registry of Patients with Rare Diseases RNPEH)* and a mechanism for the negotiation and procurement of orphan drugs.

During 2012 and 2013, efforts focused on establishing an information system for patients with RDs, acknowledging the necessity of a Colombia-specific list to facilitate the creation and management of medical records with confirmed diagnoses, the reporting of new cases to the Public Health Surveillance System (*SIVIGILA* due to its acronym in Spanish), and the establishment of a national registry for individuals with RDs. The specifics of this surveillance system will be detailed later in this paper.

*Statutory Law 1751 of 2015* further strengthened the protection of patients with RDs by recognizing them as subjects warranting special consideration and guaranteeing their access to healthcare without administrative or financial barriers*. Decree 780 of 2016* consolidated related regulations, including the mechanisms for the payment of services and technologies not financed through the Capitation Payment Unit (UPC), via ADRES (the Administrator of the different funding sources for Colombia's healthcare system).

In 2017, the payment procedure was refined through a decree stipulating that payment would only be authorized upon the patient's registration in the RNPEH. Subsequently, in 2020, provisions were issued outlining the methodology for budget allocation dedicated to the diagnosis and treatment of RDs.

The Ministry of Health and Social Protection published a guide in 2018 for the authorization of *reference centers in Colombia,* based on WHO parameters. However, the implementation of these centers has progressed slowly. At the time of writing, only four pharmacy reference centers have been officially authorized, with no centers yet designated for diagnosis and treatment.

In 2019, regulatory criteria were established for laboratory procedures to ensure quality standards within the *National Laboratory Network (RELAB),* which includes testing for the diagnosis and monitoring of rare diseases. The Ministry of Health and Social Protection issued *Resolution 1871 of 2021*, which establishes and regulates the operation of the National Board for Rare Diseases. Its key functions include serving as a central platform for the design and dissemination of public policy initiatives, defining the annual work plan, overseeing the implementation and monitoring of this plan, coordinating dialogue forums at the territorial level, and providing technical assistance to local entities concerning information dissemination and awareness campaigns.

Finally, in 2024, the Ministry of Health published the National Management Plan for Rare Diseases, which outlines specific goals and strategic directions for addressing these conditions. At the time of this publication, the plan remains under development.

## Genetic services provisions, genetic testing, medical genetics training

### Medical genetics training

As of the time of this publication, Colombia has three medical-surgical specialty programs in medical genetics. The first program was established in 2001 at the Pontificia Universidad Javeriana (PUJ) in Bogotá, with the approval of the Ministry of Health (Pontificia Universidad Javeriana – Facultad de Medicina 2024). Prior to this, the country had some training programs in human genetics, but these were structured as master's degrees rather than medical specialty programs (offered by Universidad Nacional, Universidad del Rosario and Universidad de Antioquia) (Rodas-Pérez et al. 2015).

The establishment of the medical genetics program at Javeriana University was driven by the scientific, technical, and human resources support of the Institute of Human Genetics at the same institution, alongside its strategic partnership with the Hospital Universitario San Ignacio. The Institute is recognized by the Colombian Ministry of Science and Technology as a center of research excellence, and the hospital holds high-quality accreditation and certification as a University Hospital in Colombia. Among the three medical genetics programs in the country, this program received high-quality accreditation in 2018 from the Colombian Ministry of Education and is currently undergoing the renewal process in accordance with the Ministry’s guidelines. The central curricular pillars of this program are clinical genetics, human genetics, research, teaching, and public health (Pontificia Universidad Javeriana – Facultad de Medicina 2024). To date, the program has produced 38 graduates, with 63% working in Bogotá and 37% in other regions. Notably, the southern regions of the country still exhibit a lack of presence from these graduates. Most alumni are engaged in clinical care activities within public and private healthcare institutions (75%). However, the program's comprehensive training also equips graduates to contribute to areas such as teaching and research (33%), molecular clinical laboratories (11%), and the pharmaceutical industry (12%). Importantly, at least 30% of these graduates fulfill combined professional roles.

The two other medical genetics programs in Colombia were established more recently, representing a significant step in the expansion of this specialty within the country. Common characteristics across all three programs include a three-year duration, a profesional profile encompassing clinical practice, research, teaching, and laboratory management/coordination, and dedicated clinical coursework in medical genetics and related specialties (Fundación universitaria ciencias de la salud, 2024).; ICESI 2024; Pontificia Universidad Javeriana – Facultad de Medicina 2024). Public health is a relevant component in two of the programs, while research, molecular laboratory training, and internationalization are distinctive features of the PUJ program in comparison to the others. Despite this growth, these programs remain geographically concentrated in Bogotá (PUJ and FUCS) and Valle del Cauca (ICESI). The average annual output of graduates from the three programs is eight, posing considerable challenges for building sufficient human capital, particularly in underserved regions. This centralization of specialized medical-surgical programs is a common trend throughout Colombia, as evidenced by official statistics (Ministerio de Salud de Colombia 2010).

Finally, there are broader challenges in genetics education in Colombia. There are currently no genetic counseling programs for non-physician professionals, a situation shared with many countries in the region and even globally. This gap increases difficulties not only in accessing genetic services but also in their coordination, problem-solving capacity, and support for families and patients. (Ormond et al. 2018; Paneque et al. 2024). Additionally, there is limited research and a lack of specific national guidelines on the minimum competencies in medical genetics for undergraduate health programs or other medical-surgical specialties. Each program in the country defines its own specific curricular elements for the graduate profile.

### Genetic services provisions

The formal recognition of medical genetics as a medical-surgical specialty in Colombia in 2015 led to the establishment of *the Colombian Association of Medical Geneticists and Genomic Medicine (ACMGEN).* This professional organization unites graduates and current residents from national medical genetics programs, as well as professionals who had previously obtained master's degrees in human genetics—prior to the inception of these specialty programs—and who have been officially recognized to practice medical genetics in the country through formal processes with the Ministry of Education (ACMGEN 2024).

Currently, the ACMGEN comprises 68 active members, including 42 board-certified medical geneticists, 13 medical genetics residents, and 13 honorary or associate members. To characterize this professional community, a voluntary data update survey was conducted, yielding a high response rate of 95.2% (40 out of 42 full members). Among the respondents, 82.5% (*n* = 33) are practicing in Colombia, while the remaining professionals are in other countries, including Mexico, Costa Rica, Chile, Ecuador, Canada, the United States, and Spain. This geographic distribution underscores a strong national retention of human talent in the field of medical genetics, alongside a growing international presence, particularly within Latin America, thereby facilitating the regional expansion of genomic medicine.

Among those working in Colombia, 40% are based in Bogotá and surrounding areas, while the remaining 60% are distributed across other departments including Antioquia, Valle del Cauca, Atlántico, and Santander. This territorial distribution aligns with previously reported patterns for healthcare human resources by the Colombian Ministry of Health (Ministerio de Salud de Colombia 2010). However, 21 out of the country's 32 departments (65.6%) lack local medical genetics consultation services, and 11 departments (34.4%) have no resident medical geneticists, indicating a persistent limitation in the territorial coverage of specialized genetic services.

Regarding professional practice, 57% of medical geneticists are affiliated with more than one institution. Although 62.5% report engaging in clinical activities, only 42.5% identify clinical care as their primary occupation. The remainder work in areas such as university teaching (15%), genetic diagnostics and healthcare services (12.5%), and the pharmaceutical industry (10%). While this functional diversification adds value to the field, it also reflects a limited clinical engagement, which may exacerbate existing gaps in access to specialized genetic services, a pattern that has similarly been documented in other countries in the region (Boulyjenkov 2003; Horovitz et al. 2024; Vishnopolska et al. 2018).The estimated density of medical geneticists in 2023, at a stark 0.01 per 100,000 inhabitants, reveals a significantly limited workforce even when compared to the distribution of other medical specialties (Ministerio de Salud de Colombia 2010).

In terms of infrastructure, medical genetics services are concentrated in 33 healthcare institutions, including 7 public facilities (located in Bogotá and other departments) and 26 private ones. Among these, 11 institutions hold high-quality accreditation, representing 28% of all nationally accredited healthcare institutions (ICONTEC & Ministerio de salud de Colombia 2024). This represents a positive development, considering that Colombia has only 59 high-quality healthcare institutions; however, it remains insufficient to meet the country's demands, particularly at the territorial level, where quality accreditation processes tend to be prolonged and challenging to implement. See Table 1.

**Table 1:** Distribution of medical geneticists across the departments of Colombia in 2024. Figure adapted from the Human Talent Observatory, Ministry of Health of Colombia (Ministerio de salud de Colombia 2010)

Departament	Estimated number of medical geneticists	Density per 10,000 inhabitants
Bogotá	15	0.02
Antioquia	9	0.01
Santander	3	0.01
Atlántico	2	0.01
Caldas	2	0.02
Nariño	2	0.01
Risaralda	2	0.02
Bolívar	1	Less than 1/10,000
Cesar	1	0.01
Norte de Santander	1	0.01
Tolima	1	0.01
Valle del Cauca	1	Less than 1/10,000

A remarkable finding is the improved positioning of medical genetics within the clinical setting in Colombia, particularly when compared to the perceptions reported in previous studies which highlighted significant shortcomings in medical genetics training and the availability of related services (Rodas-Pérez et al. 2015).

The expansion of medical genetics consultation services in Colombia has occurred in paralleled with the growing number of private laboratories offering molecular diagnostic testing to the general population. This trend has been notably driven by the incorporation of molecular studies into the benefits plan of *the General System of Social Security in Health (SGSSS),* positioning Colombia as an attractive market for foreign companies, investors, and domestic firms in the biomedical sector.

To characterize this landscape, a search was conducted in the Registro Único Empresarial y Social (RUES), focusing on legally registered companies in Bogotá whose economic activity falls under “Human Health Care and Social Assistance Activities,” specifically those classified as providers of diagnostic support services. The search included business registrations from January 1, 1910, to February 28, 2024 (https://www.rues.org.co/?old=true). A total of 343 companies were identified, of which 26 were selected for analysis based on their direct involvement in clinical genetics-related diagnostic services. 2 companies were excluded due to insufficient information on their official websites to determine the types of genetic tests offered.

Of the private laboratories based in Bogotá, 79.2% have their headquarters in Colombia, indicating a significant national development in molecular diagnostic capacity. The portafolio of tests offered is heterogeneous; however, it is noteworthy that 66.7% of laboratories have already implemented next-generation sequencing (NGS) technologies, and 45.8% offer chromosomal microarray analysis — both recognized as essential tools for diagnosing RDs and other conditions of interest such as cancer (e.g., somatic NGS).

A particularly relevant finding is the high prevalence of prenatal diagnostic testing (70.8%), which appears to be directly linked to the integration of these services into comprehensive maternal-perinatal care pathways within the Colombian healthcare system. Among the 17 laboratories offering prenatal diagnostics, 13 provide karyotyping and fluorescence in situ hybridization (FISH), 10 offer chromosomal microarray analysis, and 13 conduct next-generation sequencing (NGS), reflecting substantial progress in the adoption of advanced technologies for prenatal care.

This advancement is further supported by the national health system’s broad coverage of prenatal diagnostic procedures, including conventional karyotyping, FISH, array comparative genomic hybridization (aCGH), whole exome sequencing (WES), and even whole genome sequencing (WGS), provided there is a well-justified clinical indication. A notable example of this comprehensive approach is the largest prenatal diagnostic cohort reported in the country, conducted at Clínica Universitaria Colombia. In this study, López et al. analyzed 3,961 consecutive prenatal samples over a seven-year period, applying a combination of cytogenetic and molecular techniques—including karyotyping, FISH, and aCGH—to identify a wide spectrum of chromosomal abnormalities. These findings underscore the routine and systematic integration of high-resolution genomic diagnostics within both public and private healthcare settings in Colombia (López Rivera et al. 2022).

Complementing these efforts, the implementation of non-invasive prenatal testing (NIPT) has gained increasing momentum. In October 2024, Yorgene Health established the first fully integrated NIPT workflow in Colombia, in collaboration with the national company Genetix. This milestone enabled local processing of samples—eliminating the need to send them abroad—and marked a turning point in the accessibility and autonomy of prenatal genomic testing in the country. At present, at least seven laboratories nationwide offer NIPT with high sensitivity from as early as 9–10 weeks of gestation, with some expanding their services to include genome-wide screening capabilities.

Despite these advances, certain technologies remain in earlier stages of implementation. For instance, the availability of tests not yet covered by the national health benefits plan, such as preimplantation genetic diagnosis (PGD), is gradually increasing and currently offered by 16.7% of laboratories. Additionally, only one institution reported offering optical genome mapping, signaling both the emerging promise of this technology and the need to strengthen clinical awareness regarding its applications and appropriate indications.

Regarding clinical integration, only 7 laboratories (29.2%) report having an in-house specialized genetics consultation service. In practice, most genetic tests are requested by physicians in hospitals and clinics but processed externally by laboratories contracted by insurance providers, each operating under their own quality standards. This model limits the treating physician’s ability to choose the testing laboratory or to maintain close interaction with the diagnostic process. Additionally, test results are often not integrated into the patient’s electronic health record, contributing to fragmented care due to limited system interoperability and the outsourcing of services. Finally, four laboratories were identified as offering direct-to-consumer genetic testing, a trend that raises significant ethical and regulatory concerns given the absence of clear standards in Colombia regarding their use, interpretation, and clinical follow-up. This phenomenon underscores the urgent need for a national discussion on the regulatory framework and the bioethical implications of their implementation (FDA 2019).

### Therapies for patients with rare diseases

In Colombia, there is no specific legal classification for"orphan drugs."However, medications intended for the treatment of rare diseases are recognized within the health system and may or may not hold formal market authorization (i.e., sanitary registration). Such drugs can be requested under the designation of “*vital unavailable medications”* (medicamentos vitales no disponibles, MVND). *The Instituto Nacional de Vigilancia de Medicamentos y Alimentos (INVIMA),* Colombia’s national regulatory agency, is responsible for authorizing the importation of MVNDs (https://www.invima.gov.co/).

The approval process involves the individualized evaluation of each application, considering factors such as the lack of therapeutic alternatives in the country and the available scientific evidence regarding the safety and efficacy of the requested drug. According to Decree 481 of 2004, an MVND is defined as an indispensable and irreplaceable medication needed to preserve life or alleviate suffering, which is not commercially available in the country due to low profitability or is available only in insufficient quantities to meet clinical demand.

In recent years, Colombia has made notable progress in the approval and availability of targeted therapies for rare diseases, particularly in the incorporation of enzyme replacement therapies (ERTs) and other specialized pharmaceutical agents (see Table 2).

**Table 2:** Summary of the genetic testing services provided by the 24 companies included in the analysis

Test	Number of Companies Offering the Test
Preimplantation Testing	4
Prenatal Diagnosis/Screening	17
Karyotyping	13
FISH (Fluorescence In Situ Hybridization)	13
Microarrays	11
MLPA (Multiplex Ligation-dependent Probe Amplification)	11
qPCR (Quantitative Polymerase Chain Reaction)	16
Sanger Sequencing	15
NGS (Next-Generation Sequencing)	16
Optical Genome Mapping	1
PRS (Polygenic Risk Scores)	3
Direct-to-Consumer Tests	4
Somatic NGS	9
Liquid Biopsy NGS	5
Pharmacogenetics	9
Paternity Testing	8

Despite these advancements, challenges remain in the financing and reimbursement of registered orphan drugs. These are primarily related to the structure of the national health system and price regulation policies. A study analyzing MVND import requests submitted in 2016 and 2017 reported that 76% of applications were approved, reflecting a substantial institutional effort to ensure access to essential treatments. However, most patients included in these applications were enrolled in the contributory health insurance regime, revealing a structural inequity in access to health services and medications for individuals with rare diseases in Colombia (Olivares et al. 2021) Table 3.

**Table 3:** Biologics and drugs with INVIMA registration as therapeutic options for rare diseases. Consulted on 02 April 2025

Approval Date (INVIMA)	Trade Name	Active Ingredient	Indication	Marketing Authorization Holder
2010	Cerezyme®	Imiglucerase	Long-term enzyme replacement therapy (ERT) in patients with a confirmed diagnosis of non-neuropathic (type 1) or chronic neuropathic (type 3) Gaucher disease who present clinically significant non-neurological manifestations of the disease	Genzyme Corporation
2014	Feiba®	Anti-Inhibitor Coagulant Complex	Treatment and prophylaxis of bleeding and during surgical interventions in patients with hemophilia A with factor VIII inhibitor and patients with hemophilia B with factor IX inhibitor	Takeda Colombia S.A.S
2015	Fabrazyme®	Agalsidase Beta	Patients with classic Fabry disease and patients with late-onset Fabry phenotype with disease manifestations	Genzyme Corporation
2016	Vimizim®	Elosulfase Alfa	Patients with Mucopolysaccharidosis IV type A	Biomarin Colombia Ltda
2016	Naglazyme®	Galsulfase	Long-term ERT in patients with a confirmed diagnosis of Mucopolysaccharidosis VI	Biomarin Colombia Ltda
2017	Soliris®	Eculizumab	Adults and children for the treatment of Paroxysmal nocturnal hemoglobinuria, patients with hemolysis with one or more clinical symptoms indicative of high disease activity, regardless of transfusion history. Atypical hemolytic uremic síndrome	Astrazeneca Colombia S.A.S
2017	Elaprase®	Idursulfase	ERT in patients with Mucopolysaccharidosis II	Takeda Colombia S.A.S
2017	Adynovate®	Factor VIII	Children and adults with hemophilia A	Takeda Colombia S.A.S
2018	Replagal®	Agalsidase Alfa	Long-term (ERT) in patients with Fabry disease	Takeda Colombia S.A.S
2018	Aldurazyme®	Laronidase	Long-term ERT in patients with a confirmed diagnosis of Mucopolysaccharidosis I	Genzyme Corporation
2018	Cordela®	Eliglustat Tartrate	Long-term treatment of adult patients with type 1 Gaucher disease (GD1) who are poor, intermediate, or extensive CYP2D6 metabolizers	Sanofi B.V
2019	Galafold®	Migalastat	Long-term treatment of adults and adolescents aged 12 years and older with a confirmed diagnosis of Fabry disease (alpha-galactosidase A deficiency) and with mutations amenable to treatment	Pint Pharma International
2019	Firazyr®	Icatibant Acetate	Symptomatic treatment of acute attacks of hereditary angioedema (HAE) in adults, adolescents, and children over 2 years old with C1 esterase inhibitor deficiency	Takeda Colombia S.A.S
2019	Spinraza®	Nusinersen	Patients with genetically confirmed 5q spinal muscular atrophy with 2 or more copies of the SMN2 gene and functional motor assessment using a validated scale: type 0 or 1 (Werdnig-Hoffman) under 6 months, or type 2 and 3 up to 6 years old, i.e., not yet 7 years old	Biib Colombia S.A.S
2020	Bantace®	Imiglucerase	Long-term ERT in pediatric and adult patients with a confirmed diagnosis of non-neuropathic type 1 or chronic neuropathic (type 3) Gaucher disease who present clinically significant non-neurological manifestations of the disease	Isu Abxis Co.,Ltd
2020	Miozyme®	Alglucosidase Alfa	Long-term ERT for patients with a confirmed diagnosis of Pompe disease (acid alpha-glucosidase deficiency)	Sanofi B.V
2020	Strensiq®	Asfotase Alfa	ERT in patients under 12 years of age with hypophosphatasia to treat bone manifestations of the disease demonstrated by clinical, paraclinical, and genetic diagnosis	Astrazeneca Colombia S.A.S
2020	Revestive®	Teduglutide	Short bowel syndrome (SBS) in patients aged 1 year and older	Takeda Colombia S.A.S
2020	Takhzyro®	Lanadelumab	Prevention of recurrent hereditary angioedema (HAE) attacks type I or II in patients aged 2 years and older	Takeda Colombia S.A.S
2021	Cuchel®	Trientine Hydrochloride	Patients with Wilson's disease who are intolerant to D-penicillamine	Msn Laboratories Private Limited
2022	Cubrital®	Trientine Hydrochloride	Patients with Wilson's disease who are intolerant to D-penicillamine	Alatin Resources Inc
2022	Cuprior®	Trientine Hydrochloride	Treatment of Wilson's disease in adults, adolescents, and children aged 5 years and older who are intolerant to D-penicillamine	Orphalan S.A
2022	Metopirone®	Metyrapone	Indicated either as a diagnostic test for ACTH insufficiency and in the differential diagnosis of ACTH-dependent Cushing's syndrome, or for the management of patients with endogenous Cushing's syndrome	Hra Pharma Rare Diseases
2022	Exabant®	Icatibant Acetate	Symptomatic treatment of acute attacks of hereditary angioedema (HAE) in adults, adolescents, and children over 2 years old with C1 esterase inhibitor deficiency	Exeltis S.A.S
2023	Elizaria®	Eculizumab	Patients with Paroxysmal nocturnal hemoglobinuria, Atypical hemolytic uremic síndrome	Generium S.A
2023	Cuprexit®	Trientine Hydrochloride	Patients with Wilson's disease who are intolerant to D-penicillamine	Liminal Therapeutics
2023	Crysvita®	Burosumab	X-linked hypophosphatemia	Ultragenyx Colombia
2023	Tegsedi®	Inotersen Sodium	Polyneuropathy of hereditary transthyretin-mediated amyloidosis in adults at stage 1 or 2, documented by genotyping and tissue biopsy, who have not undergone liver transplantation	Ptc Therapeutics Colombia S.A.S
2024	Tritixor®	Trientine Hydrochloride	Wilson's disease who are intolerant to D-penicillamine	Vexxor Medical Inc
2024	Willobant®	Icatibant Acetate	Symptomatic treatment of acute attacks of hereditary angioedema in adults, adolescents, and children over 2 years old with C1 esterase inhibitor deficiency	Willow Pharma S.A.S
2015	Vpriv®	Velaglucerase Alfa	Long-term (ERT) in pediatric and adult patients with type 1 Gaucher disease	Takeda Colombia S.A.S
2023	Evrysdi®	Risdiplam	Treatment in patients with genetically confirmed 5q (SMA) with 2 or more copies of the SMN2 gene and functional motor assessment using a validated scale: type 1 or type 2 and 3 in patients up to 25 years of age	F. Hoffmann—La Roche Ltd
2024	Zolgensma®	Onasemnogene Abeparvovec	Pediatric patients under 2 years of age with spinal muscular atrophy (SMA) with biallelic mutations in the survival motor neuron 1 (SMN1) gene	Novartis de Colombia
2025	Luxturna®	Voretigene Neparvovec	Adult and pediatric patients with vision loss due to inherited retinal dystrophy associated with confirmed biallelic RPE65 mutation and who have sufficient viable retinal cells	Novartis Pharma AG

## Genetic epidemiology

### Newborn screening

Newborn screening (NBS) is widely recognized as a critical tool for the prevention and management of RDs and birth defects (BD). In Colombia, the national NBS policy was first introduced in the year 2000, initially focusing exclusively on the detection of congenital hypothyroidism through umbilical cord blood simples. (Ministerio de Salud y protección Social de Colombia 2000).

The formal recognition of rare diseases (RDs) as a public health priority in Colombia was established through *Law 1392 of 2010.* This acknowledgment is particularly significant in the historical context of newborn screening (NBS) development in the country, as many rare conditions can be detected early through such programs. Early identification enables timely intervention, reduces mortality and long-term disability, and contributes to the more efficient use of healthcare resources.

Initial regulatory efforts included the publication of the *Comprehensive Care Guidelines for the Healthy Newborn and the Guidelines for the Detection of Congenital Anomalies* in 2013. These documents laid the foundation for national screening policies by incorporating auditory, metabolic, and visual screening components into the routine care of newborns (Ministerio de Salud y Protección Social de Colombia 2013).

Subsequently, *Law 019 of 2015* was enacted to establish the structural foundation of the national newborn screening (NBS) program, including the creation of a dedicated Directorate within the National Institute of Health (Instituto Nacional de Salud, INS) to serve as the governing body. This legislative effort culminated in the passage of *Law 1980 of 2019,* which formally institutionalized the NBS Program. The law provided a regulatory framework for the early detection of congenital anomalies, the management of biological samples, and the coordination among healthcare system stakeholders, thereby reinforcing the operational and normative structure necessary for nationwide implementation (Congreso de Colombia., 2019).

A significant milestone in the program’s development occurred in 2018 with the definition of *the Integrated Health Care Pathways (Rutas Integrales de Atención en Salud, RIAS),* which established guidelines for health promotion and maintenance across different age groups, with a particular emphasis on the maternal and perinatal population. These pathways incorporated screening strategies for auditory and visual impairments, as well as for congenital heart defects (Ministerio de Salud y Protección Social 2018).

In 2024, the Ministry of Health and Social Protection issued updated technical guidelines for the national NBS program which are mandatory for all stakeholders involved in the management and direct care of newborns (Ministerio de Salud y Protección Social 2024).

Despite Colombia’s comprehensive regulatory framework, significant implementation gaps remain, even for congenital hypothyroidism (CH), the first condition included in the NBS program decades ago. As of 2024, the estimated NBS coverage for CH ranges between 75–90%, comparable to levels reported in Mexico and Brazil. However, recent reports indicate that between 2014 and 2024, Colombia’s coverage has only increased by approximately 25%, showing limited progress (Therrell et al. 2024). The documented challenges include delays in treatment initiation following diagnosis notification, incomplete data entry in national databases, and lack of standardized protocols for sample collection—even within Bogotá (Pineda-Sanabria et al. 2023).

Furthermore, the implementation of screening for metabolic disorders—despite being included in clinical guidelines and national policy—remains largely limited to pilot programs in selected clinics and laboratories. This stands in contrast to other countries in the region that have achieved broader implementation of screening for this group of diseases (Therrell et al. 2024). In conclusion, as of 2024, Colombia’s active newborn screening program includes tests for congenital hypothyroidism, complex congenital heart disease, hearing impairment and visual disorders.

### Surveillance of BD

Colombia has developed a robust BD surveillance system over the past 20 years, aimed at understanding their impact on public health, identifying epidemiological trends, and strengthening prevention and care strategies. This effort has been driven primarily by programs based in Bogotá and Cali, which have evolved from early hospital-based surveillance models into more comprehensive systems that integrate clinical records with data from the Public Health Surveillance System (Sistema Nacional de Vigilancia en Salud Pública, SIVIGILA).. *The Bogotá Congenital Malformations Surveillance Program (BCMSP)* began in 2001 by monitoring a single hospital and, with support from the District Health Secretariat, successfully expanded to cover over 90% of births in the city. In Cali, a similar program was established in 2010 and following the integration of the Municipal Health Secretariat in 2016, significantly enhanced its reach and diagnostic accuracy. Both programs have successfully integrated into international surveillance networks, such as the *Latin American Collaborative Study of Congenital Malformations (ECLAMC),* and the *International Clearinghouse for Birth Defects Surveillance and Research (ICBDSR)* (ICBDSR 2015) thereby contributing to regional efforts in monitoring, research, and prevention of BD (ECLAMC 2024). This integration has enabled their data to contribute to the global understanding of these conditions, enhancing international knowledge on their prevalence, risk factors, and patterns of occurrence (OPS 2020).

Since their implementation, these programs have monitored over 1.28 million births in Bogotá and Cali, identifying approximately 25,000 cases of BD. The data collected have been instrumental in determining the prevalence of these conditions, identifying associated risk factors—such as advanced maternal age and exposure to teratogenic agents—assessing the impact of public health emergencies, including the Zika virus epidemic. Moreover, these findings have facilitated the implementation of primary prevention strategies, including food fortification with folic acid, the promotion of adequate prenatal care and the strengthening of early detection practices.

These surveillance efforts have also contributed to improved referral and follow-up for patients with BD, enhancing their access to specialized medical services and timely treatments. One of the most significant achievements of these programs has been the successful collaboration between academic institutions and public health authorities, allowing surveillance efforts not only to capture cases but also to generate actionable knowledge for maternal and child health policymaking.

Despite these advances, BD surveillance in Colombia continues to face multiple challenges. Barriers remain in expanding coverage to rural and vulnerable populations where access to healthcare systems is limited. Additionally, there is a need to improve the quality of data recorded in SIVIGILA through enhanced training of healthcare personnel and the implementation of innovative technological tools that enable more efficient and accurate data analysis.

The incorporation of artificial intelligence and predictive modeling could further optimize the identification of epidemiological patterns and support data-driven decision-making in public health. Looking ahead, it is essential not only to sustain but also to evolve the surveillance system toward a more integrated and accessible model—one that ensures early detection, comprehensive care, and appropriate follow-up for children with BD, ultimately improving their quality of life and that of their families.

### Surveillance of rare diseases

Between 6,000 and 7,000 RD have been identified worldwide. Colombia established the foundational pillars for the information system of these patients in 2012 through a national decree. Later, in *Resolution 3681 of 2013,* the technical requirements for reporting the Patient Census information were defined. The first report recorded 13,173 patients in 2013, with 53.96% of them being female, a median age of 28 years, and the most common diseases being Congenital Factor VIII Deficiency, Myasthenia Gravis, Von Willebrand Disease, Short Stature due to Growth Hormone Deficiency, and Bronchopulmonary Dysplasia. At that time, it was decided that updates would occur every two years. To date, the registry has been updated three times under different resolutions, with the most recent being Resolution 023 of 2023, which includes a total of 2,247 diseases. (Ministerio de Salud y Protección Social 2024).

The National Rare Diseases Technical Committee has reviewed the procedure for reporting RDs in the SIVIGILA system with the aim of improving the representativeness of cases in the RNPEH. This is the national and continuous registry of basic data that identifies individuals diagnosed with rare diseases in the country, managed by the Ministry of Health and Social Protection. It started in 2013 by registering historical cases through the census and is continuously updated through the reporting of new cases to SIVIGILA at the National Institute of Health (INS)(Ministerio de Salud y Protección Social 2024).

Through *Resolution 946 of 2019,* it is established that the Health Benefit Plan Administrators (EAPB) and healthcare service providers must report cases according to the Public Health Surveillance Protocol for RD of the INS (Instituto nacional de salud 2024). According to the INS, case notifications have shown a continuous increase, reaching the highest peak in 2019 with 12,714 reported individuals. However, in 2020, there was a decline in the reporting of these cases, likely related to the SARS-CoV-2 pandemic. The latest report as of April 2024 recorded 84,175 individuals diagnosed with RDs (26). According to the National Management Plan for Rare Diseases in 2022, 89.2% of the cases were reported as living, and 74.8% had been reported by EAPBs from the private sector, followed by public EAPBs (20.3%). Additionally, 88.3% of the cases had a disability status.

57.2% of patients with RDs are female, and their age distribution follows a bimodal pattern: they are primarily between the ages of 10 to 14 years and from 55 to 59 years. Nervous system diseases (24.4%) are the leading cause in adults aged 55 to 59 years, followed by hematopoietic and immunological diseases (19.1%). In contrast, congenital and chromosomal diseases (15.8%) are more common among individuals aged 20 to 24 years, while endocrine and metabolic diseases are most prevalent in those aged 10 to 14 years (28). The most frequently reported diseases are: multiple sclerosis with 5,166 cases, Von Willebrand disease with 3,951 cases, and congenital factor VIII deficiency with 3,328 cases (Instituto nacional de salud 2024; Ministerio de Salud y Protección Social 2024).

It is important to highlight that most reported cases are concentrated in the major urban centers, such as Antioquia (20.6%), Bogotá D.C. (12.8%), Valle del Cauca (11.6%), Cundinamarca (4.3%), and Santander (3.2%). This distribution suggests a potential underdiagnosis and underreporting in rural and hard-to-reach areas, where no cases have been reported according to the data published by the INS (0% of the accumulated proportion of notified individuals) (Ministerio de Salud y Protección Social 2024).

As part of epidemiological surveillance and strengthening the diagnosis of RDs in Colombia, the Ministry of Health and Social Protection has established mechanisms to allow the registration of laboratories conducting tests or assays related to this group of pathologies in the National Laboratory Registry (RELAB)(Ministerio de Salud y Protección Social 2019). This registration is a key step in ensuring the traceability and quality of diagnostic tests at the national level. Each test must be properly registered and accredited by the National Accreditation Organization of Colombia (ONAC) (Ministerio de Salud y Protección Social 2019; ONAC 2024), which has been the official accreditation body in the country since 2008. In line with these policies, one of the national goals projected for 2031 is that 100% of the departmental and district public health laboratories will be trained to provide technical assistance in the registration of clinical laboratories conducting diagnostic tests this group of diseases in the RELAB. This objective aims to expand the installed capacity and improve equity in access to diagnosis across the entire national territory (Ministerio de Salud y Protección Social 2019).

### Genomic medicine

Genomic medicine in Colombia began in the 1990 s when molecular biology techniques were introduced to the laboratories of a group of universities, including the National University, the University of Rosario, and the Javeriana University. These early academic efforts led to the first genotyping, primarily of patients with diseases of national interest, such as cystic fibrosis and Huntington's disease. Building on these developments, the technology was transferred to private laboratories offering services like sequencing, panels, or evaluation of specific mutations, which are reimbursed by the health system, as previously described. This characteristic sets Colombia apart from the rest of the region, where molecular tests are difficult to obtain and must be paid with the affected families own resources.

The field of human genomics and its integration into healthcare systems in the country has shown significant progress. Key achievements include: 1) the consolidation of research groups and centers of excellence in human genetics; 2) the expansion of medical genetics programs in the country; 3) the development of technological infrastructure in genomics laboratories focused on clinical practice; 4) the evolution of regulations to facilitate access to genetic diagnostic technologies and services; and 5) the formation of two key scientific associations in human and medical genetics, such as the Colombian Association of Human Genetics (ACGH) and the Colombian Association of Medical Genetics and Genomic Medicine (ACMGen), members of the Colombian Association of Scientific Societies (ACGH 2024; ACMGEN 2024).

Additionally, three recent initiatives in which Colombia actively participates are highlighted. The first is *LatinOMICS*, an alliance driven by scientists, academic institutions, and private organizations and the *Latin American Network of Human Genetics (RELAGH),* with the goal of promoting research in genomics and omics sciences in Latin America and the Caribbean. This initiative is advancing towards personalized medicine by improving diagnoses, treatments, and disease prevention through genetic analysis. It aims to position the region as a leader in this field by 2050, establishing genomic databases and promoting research in pharmacogenomics, RDs and chronic conditions (Consultorsalud 2024).

The second initiative is the creation of Colombia’s *first genomic and epigenomic bank*, announced in November 2024 and led by the National Institute of Health, with support from the Ministries of Health and Science. The program, named Genoma Insignia Colombia, aims to collect genetic information from 10,000 Colombians to promote precision public health, facilitating the prediction, prevention, and diagnosis of diseases. This repository will enable a better understanding of the impact of environmental factors and lifestyles on the population's genetics. With an initial investment of 1.2 million USD, the initiative seeks to ensure scientific data sovereignty and its ethical use, while also prioritizing the well-being of vulnerable communities and promoting innovative technologies in public health (Instituto nacional de salud 2024.).

Finally, a third initiative has been taking shape since 2023. The United Kingdom's National Institute for Health and the World Health Organization held an event in 2023 focused on education and training in genomics. By 2024, participants from Latin America were invited to form a regional and global network *(GGNET)* aimed at promoting collaborative work in this field, avoiding duplication and isolated efforts.

However, there are significant challenges in integrating genomics into health systems. These include: Improving the interoperability of medical records and developing robust information systems that facilitate access to genomic data; Generating local knowledge that demonstrates the clinical utility of genomic data for decision-making in healthcare; Including decision-makers in health services, such as the Ministry of Health and insurance providers, to reduce inequities in access to genomic-based health tools; Training human talent in bioinformatics; Establishing clear ethical and regulatory guidelines on the management of genomic data, including informed consent, data use, and privacy—particularly in a diverse society such as Colombia; and Securing the necessary funding for this integration and for research that strengthens the generation of local data.

### Patient organizations

In accordance with the Colombian Constitutional Charter and other legislative mechanisms, citizen participation mechanisms in Colombia have played a decisive role in strengthening the country’s healthcare system (Constitución política de Colombia 1991). A clear example of this is the presence of various organizations and foundations of patients with RDs, which have played a pivotal role in the development of public policies, regulatory frameworks, and the design and implementation of care pathways for these patients and their families. Moreover, they have contributed significantly to the creation of support networks for caregivers and patients alike. This section highlights two patient organizations with established trajectories and measurable impact in the country.

### FECOER

*The Colombian Federation of Rare Diseases (FECOER)* began its activities in 2011 as a response to “a need for independent associations to come together to address common problems and to establish mutual advocacy.” It comprises 39 foundations and patient associations with various RDs, including neurodegenerative conditions, short stature, inborn errors of metabolism, hematologic diseases, genodermatoses, among others (FECOER 2024.)

Its main achievements can be summarized as follows:

• Raising awareness about RDs in Colombia by promoting education and sensitization among society and healthcare professionals.
• Advocating for the rights of patients with RDs, driving improvements in public policy to ensure access to treatments and healthcare services.
• Creating support networks for patients and families, facilitating the exchange of information and experiences.
• Promoting research projects in the field of rare diseases, aiming to support improved diagnostics and treatments.
• Establishing international collaborations through alliances with global organizations and other federations to share resources, knowledge, and experiences in RDs management.
• Organizing conferences, workshops, and meetings to train healthcare professionals and raise awareness in the community about the challenges and needs of people with RDs.

### ENHU

The *Interinstitutional Observatory of Rare Diseases (ENHU)* aims to “efficiently and harmoniously coordinate private efforts from various actors directly or indirectly involved in the healthcare system, with the common goal of designing, proposing, and influencing policies to improve the quality of care for patients with rare diseases” (ENHU 2024).

It is a non-profit association that encompasses 36 foundations and patient associations with a wide range of conditions. Among these are the Colombian Association of Patients with Lysosomal Storage Diseases (ACOPEL), the Colombian Association of Patients with Cornelia de Lange Syndrome (CdLS), and the Colombian Foundation for Cystic Fibrosis and Other Respiratory Diseases (FIQUIRES).

ENHU, working transversally with different entities and organizations involved in the care of patients with RDs, has created spaces for awareness and inclusion. Each year, it organizes an event to commemorate Rare Disease Day, in collaboration with public and private sector stakeholders, academia, patient associations, caregivers, media, and others.

## Conclusions

Medical genetics in Colombia has undergone a significant evolution as a medical discipline, establishing itself primarily in the areas of healthcare service delivery, public health and academic settings. Noteworthy progress has been achieved in public health (notably in the surveillance of BDs and RDs), the growth of medical and surgical training programs (although still insufficient to meet demand), and the development of clinical and diagnostic laboratory services. However, important gaps and challenges remain, particularly in the integration of genomics into the healthcare system for complex conditions, the generation of local knowledge to inform clinical decision-making, interoperability to ensure access to and appropriate use of genomic data, and the implementation of basic preventive tools such as newborn screening.

Colombia possesses robust regulatory frameworks and public policy development in the field; nevertheless, the operationalization and implementation of these regulations remain a significant challenge. Over the past 20 years, the development of medical genetics has been substantial, particularly in terms of access to technologies and services, including medications for patients with RDs. The structure of the Colombian healthcare system has facilitated such advancements. Citizen participation mechanisms have been especially notable in promoting inclusion and guaranteeing the right to health. Although there are no specific metrics on public literacy, the efforts made by both patient organizations and professional associations in genetics and other disciplines over the past two decades have been instrumental in raising awareness and visibility.

Nonetheless, global sustainability challenges within the healthcare system directly affect the care of patients with rare and other high-cost conditions. These include gaps in access to timely diagnoses, continuity of care, availability, training of sufficient and qualified human resources for multidisciplinary care, and the assurance of quality and accessibility—particularly in rural areas. This includes access to genetic consultations, infrastructure, quality and reliability of diagnostic testing.

Recent policies and national goals—such as universal coverage through RELAB, the national plan for rare diseases, and the strengthening of Integrated Health Care Routes (RIAS)—provide concrete frameworks for consolidating medical genetics as a strategic component of public health. Furthermore, Colombia’s active participation at the regional level in Latin America, through collaborative efforts to define standards, support joint initiatives, and coordinate actions, is emerging as a strategic imperative to strengthen both medical genetics and public health across the region.

## Limitations

This article presents a comprehensive and updated overview of the progress and challenges of medical genetics and genomic medicine in Colombia; however, it is not without limitations. The article provides a broad and detailed overview of the development of medical genetics in Colombia; however, its primarily descriptive nature limits the ability to establish causal relationships or quantitatively assess the impact of implemented policies.

## Data Availability

No datasets were generated or analysed during the current study.

## Declarations

The authors used **ChatGPT Plus** to support language editing and formatting during the preparation of this manuscript. These AI-assisted improvements were limited to enhancing readability, grammar, and structure, and did **not** involve generative editorial work or autonomous content creation. All final content reflects the authors’ original work, and full human accountability is maintained.

### Human or animal

This article does not contain any studies with human or animal subjects performed by the any of the authors.
